# Effects of personalized vitamin D_3_ on inflammation in colorectal cancer patients: a randomized trial

**DOI:** 10.1038/s41416-025-03333-6

**Published:** 2026-01-08

**Authors:** Tafirenyika Gwenzi, Alexander N. R. Weber, Kira Trares, Tomislav Vlaski, Marija Slavic, Sha Sha, Edelmann Dominic, Reiner Caspari, Bettine Bilsing, Harald Fischer, Cristina-Maria Fernandes-Almeida, David Czock, Ben Schöttker, Hermann Brenner

**Affiliations:** 1 Division of Clinical Epidemiology of Early Cancer Detection, German Cancer Research Center (DKFZ) Heidelberg, Heidelberg, Germany; 2 Division of Primary Cancer Prevention, German Cancer Research Center (DKFZ) Heidelberg and National Center for Tumor Diseases (NCT), NCT Heidelberg, a partnership between DKFZ and University Hospital Heidelberg, Heidelberg, Germany; 3 Medical Faculty Heidelberg, Heidelberg University, Heidelberg, Germany; 4 Institute of Immunology, Department of Innate Immunity, University of Tübingen, Tübingen, Germany; 5 iFIT–Cluster of Excellence (EXC 2180) “Image-Guided and Functionally Instructed Tumor Therapies”, University of Tübingen, Tübingen, Germany; 6 CMFI–Cluster of Excellence (EXC 2124) “Controlling Microbes to Fight Infection”, University of Tübingen, Tübingen, Germany; 7 German Cancer Consortium (DKTK), Partner Site Tübingen, a partnership between DKFZ and University Hospital Tübingen, Tübingen, Germany; 8 Division of Biostatistics, German Cancer Research Center (DKFZ) Heidelberg, Heidelberg, Germany; 9 Clinic Niederrhein, Bad Neuenahr-Ahrweiler, Germany; 10 Clinic Bad Salzelmen, Schönebeck, Germany; 11 Clinic Rosenberg, Bad Driburg, Germany; 12 Rehabilitation Centre Oberharz, Braunschweig-Hannover, Germany; 13 Department of Clinical Pharmacology and Pharmacoepidemiology, University Hospital Heidelberg, Heidelberg, Germany; 14 German Cancer Consortium (DKTK), DKFZ, core center Heidelberg, Heidelberg, Germany; 15 Network Aging Research, Heidelberg University, Heidelberg, Germany

**Keywords:** Colorectal cancer, Prognostic markers

## Abstract

**Background:**

Low vitamin D status and inflammation are associated with poor prognosis among colorectal cancer (CRC) patients. We assessed the efficacy of personalized vitamin D_3_ supplementation (VIDS) for reducing inflammation in patients with low vitamin D status.

**Methods:**

In an ongoing randomized double-blind, placebo-controlled trial in Germany, CRC patients who underwent surgery in the past year and had serum 25-hydroxyvitamin D levels < 60 nmol/L were randomly assigned to either a personalized loading dose of VIDS, followed by a maintenance dose of 2000 IU/day or a placebo for 12 weeks. Changes in serum interleukin-6 (IL-6), interferon-gamma (IFN-γ), and matrix metalloproteinase (MMP-1) were compared at the end of trial among 126 patients (65 in the placebo and 61 in the intervention group).

**Results:**

The VIDS group exhibited 39.3% reduction in IL-6 levels compared to the placebo group (95% CI: −54.9% to −18.2%; *p* = 0.001). The reductions observed in IFN-γ and MMP-1 due to VIDS were not statistically significant (−6.7%; *p* = 0.69 and −5.4%; *p* = 0.23, respectively).

**Conclusion:**

In CRC patients with low vitamin D status, VIDS reduces serum IL-6, a pro-inflammatory biomarker associated with poor prognosis. Further research should explore a potential supportive therapeutic role of VIDS in managing inflammation and improving CRC outcomes. [Words: **200**].

## Introduction

Colorectal cancer (CRC) is a leading cause of cancer morbidity and mortality globally, accounting for over 1.9 million new cases and more than 900,000 deaths per year [1]. Vitamin D insufficiency and deficiency are common in CRC patients, with lower levels of serum 25-hydroxy-vitamin D (25(OH)D)—widely acknowledged as a marker of vitamin D status—associated with increased mortality [2]. While routine clinical assessment of vitamin D deficiency is not commonplace in the management of CRC patients, there is growing support for screening and subsequent normalization of 25(OH)D levels through vitamin D_3_ supplementation (VIDS) to potentially enhance prognosis [3]. Despite limited evidence from randomized controlled trials (RCTs), a recent meta-analysis indicated a noteworthy 35% improvement in progression-free survival among CRC patients receiving VIDS [4]. Additionally, VIDS has been associated with potential benefits in enhancing chemotherapy efficacy, mitigating chemotherapy-induced adverse effects, and improving health-related quality of life (HRQoL) among CRC patients [5].

Calcitriol (1,25-dihydroxycholecalciferol), the most active form of vitamin D, operates through vitamin D receptors (VDRs) expressed ubiquitously in various tissues, including intestines and immune cells. Although the precise mechanisms underlying VIDS-related enhancement of CRC outcomes remain elusive, preclinical studies suggest a role for calcitriol in modulating inflammatory processes [6]. Elevated circulating pro-inflammatory biomarkers have been associated with tumor growth, metastasis, and mortality in cancer patients [7]. A recent meta-analysis of RCTs in patients with cancer and precancerous lesions demonstrated a reduction in tumor necrosis factor-α (TNF-α), interleukin-6 (IL-6), and C-reactive protein (CRP) levels following VIDS [8]. However, previous trials may have underestimated the true effects of VIDS by administration of uniform VIDS doses without considering critical factors such as baseline vitamin D status, body mass index (BMI), and dosage regimen (high-dose bolus vs. low-dose daily). Supplementation seems most advantageous for individuals with vitamin D deficiency, suggesting that targeted VIDS striving to achieve and maintain sufficient 25(OH)D blood levels may be most effective. We aimed to evaluate the impact of personalized oral VIDS inflammatory response in patients with CRC and low vitamin D status. We hypothesise that VIDS would reduce blood-based pro-inflammatory biomarker levels.

## Materials and methods

### Study design and participants

**Fig. 1:**
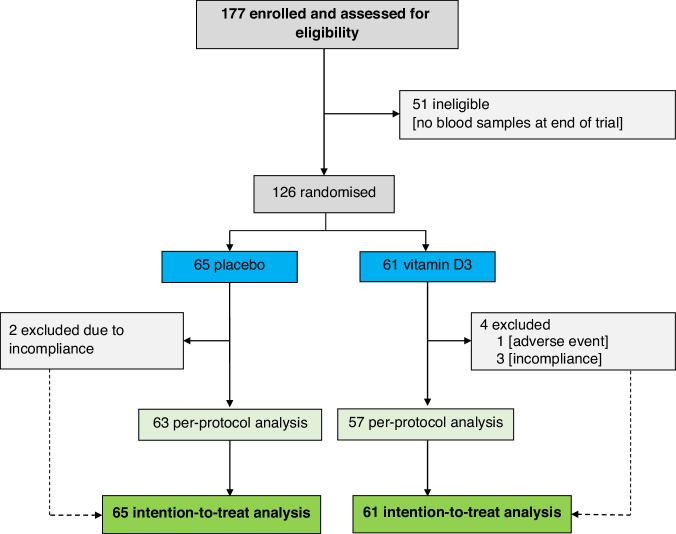
Flow diagram of participant selection and analysis. Of 177 participants assessed for eligibility, 126 were randomized to placebo (*n* = 65) or vitamin D3 (*n* = 61); 51 were excluded prior to randomization due to absence of blood samples at trial completion. In the placebo group, two participants were excluded because of non-compliance, leaving 63 for per-protocol analysis. In the vitamin D3 group, four participants were excluded (one adverse event and three due to non-compliance), leaving 57 for per-protocol analysis. All randomized participants were included in the intention-to-treat analysis.

Our study is based on data from the ongoing VICTORIA trial (German Clinical Trials Register, EudraCT-No: 2019-000502-30; DRKS00019907, website https://www.bfarm.de/EN/BfArM/Tasks/German-Clinical-Trials-Register/_node.html). Trial design details of the VICTORIA trial have been previously reported [9] (also see Supplementary Methods for more details). This ancillary study included 126 patients recruited between 23 September 2020 and 19 July 2023 who completed the study until 22 November 2023 at the latest (Fig. 1). The study adheres to the tenets of the Declaration of Helsinki (latest amendment) and was approved by the Ethics Committee of the State Chamber of Medicine in Rheinland-Pfalz (approval #: 2020-14854-AMG). All participants provided written informed consent.

### Patient and public involvement

The choices of the trial intervention and the trial end-points have been discussed with the Deutsche ILCO e.V. (a patient advocacy group for CRC). The results of the study will be disseminated to the public through conference presentations and social media.

### Intervention and control arms

Based on a computer-generated randomization list, participants were randomly assigned in a 1:1 ratio to the VIDS or placebo group. Patients and study staff were masked to the group assignment (double-blind trial). During the first 11 days, a personalized loading dose based on the 25(OH)D level and BMI at screening was administered as 20,000 IU/day or 40,000 IU/day. The personalized loading dose was calculated based on the equation reported by Jansen et al., which targets the optimal 25(OH)D levels of 75–100 nmol/L [10]:

$${{{\rm{Loading\; dose}}}}=165{{{\rm{x\; BMI}}}}[{{{\rm{kg}}}}/{{{\rm{m}}}}2]{{{\rm{x}}}}(70-{{{\rm{baseline}}}}25({{{\rm{OH}}}}){{{\rm{D\; level}}}}[{{{\rm{nmol}}}}/{{{\rm{L}}}}]).$$

Following a personalized loading dose, a maintenance dose of 2000 IU/day was administered until end of trial week 12. In the control arm, patients received placebo in the same schedule as in the intervention arm.

### Laboratory methods

Blood samples were collected at three distinct time points: baseline (BL), visit 1 on trial days 12–21 (i.e., the end of the loading dose phase and the end of the rehabilitation clinic stay, designated as FU1), and visit 2 at trial weeks 13–16 (i.e., the end of the maintenance dose phase and the end of the trial, designated as FU2). Blood samples were sent to the study center and stored at −80 °C.

#### Serum 25(OH)D measurements

Serum 25(OH)D was measured at BL, FU1, and FU2 in rehabilitation clinic laboratories using the LIAISON® 25 OH VITAMIN D TOTAL chemiluminescent immunoassay of DiaSorin, Saluggia, Italy. Based on the specifications of the manufacturer, the detection range is 10.0–375.0 nmol/L, while the intra-assay and inter-assay coefficients of variation (CV) are 5.4% and 10.6%, respectively.

#### Serum inflammatory biomarker measurements

Inflammatory biomarkers were assessed by the Olink Target 96 Inflammation panel at BL and FU2 from 15 μl of serum extracted from aliquots that had been thawed for the first time (see the full list of 92 biomarkers in Supplementary Table 1). Serum biomarker concentrations are reported as Normalized Protein eXpression (NPX) values, a relative protein quantification based on the log_2_ scale. We also measured the absolute IL-6 serum biomarker concentrations (in pg/mL) using the Olink Flex panel. Normalization of raw data was conducted to adjust for technical variations in the biomarker assays using the bridging procedure as recommended by Olink®. The Olink panels are based on a proximity extension assay technology (PEA) [11]. The average intra-assay and inter-assay CVs for the relative and absolute protein measurements were <18% at both BL and FU2.

### Outcomes

Following a pre-defined statistical analysis plan, our analyses were based on two approaches [1]: Confirmatory analysis to assess the effects of VIDS on IL-6, interferon-gamma (INF-γ) [12] and matrix metalloproteinase-1 (MMP-1) [13], which were a priori selected based on evidence on their diagnostic and prognostic value in CRC patients and [2] Exploratory analysis to assess the effects of VIDS on the other remaining biomarkers. The effect measure was the mean change in serum NPX biomarker levels between BL and FU2.

Results about the 25(OH)D efficacy outcomes and safety outcomes have been previously reported in an interim analysis with a lower sample size [14]. In this analysis, the mean 25(OH)D levels, the change in 25(OH)D levels, and the proportion of subjects exhibiting inadequate 25(OH)D levels (i.e., levels <50 nmol/L) in the intervention and placebo groups at BL, FU1, and FU2 were presented with their respective 95% CIs.

### Statistical analyses

This was an ancillary analysis of the VICTORIA trial. The sample size and randomization were determined based on the objectives of the parent trial. Therefore, a formal power calculation was not feasible, as the ancillary hypothesis and corresponding effect size were not part of the original trial design. The following patient characteristics were used to describe the study population at BL: serum 25(OH)D, IL-6, INF-γ, and MMP-1 concentrations, age, sex, cancer stage at diagnosis, time since diagnosis, time since surgery, previous chemotherapy, previous radiotherapy, comorbidities, BMI, smoking status, alcohol consumption, physical activity, and frailty status.

The main outcome results were based on the intention-to-treat (ITT) analysis, which included all 126 randomized patients (65 in the placebo group and 61 in the VIDS group). For IL-6, INF-γ, and MMP-1, within-study-arm mean NPX changes of the serum levels from BL to FU2 were presented with their respective 95% confidence intervals (CIs). Furthermore, the percentage actual between-study-arm mean difference in the biomarker serum concentrations between the placebo and intervention groups at FU2 was calculated as follows:

Percentage Actual Mean Difference at FU2 = (2^Mean NPX Difference^ − 1) × 100%.

We also estimated the effect of VIDS on the serum concentrations of IL-6, INF-γ, and MMP-1 from BL to FU2 using univariable and multivariable linear regression models. The linear regression models used the NPX biomarker values as the dependent variable, while the independent variable was the intervention group (placebo or VIDS). Denoting the coefficient of VIDS as β, the estimated change in the biomarker serum concentrations due to VIDS was calculated as follows:

Estimated Percentage Change from BL to FU2 = (2^β^ − 1) × 100%.

For the confirmatory analysis, the Bonferroni correction for multiple testing was applied, i.e., two-sided *p* values < 0.0166 were considered statistically significant. In addition, per-protocol (PP) and sensitivity analyses were also conducted. The PP analysis excluded five participants who exhibited less than 80% compliance with the trial medication, as well as one participant in the intervention arm who discontinued treatment due to hypercalcemia (see Fig. 1). In the sensitivity analyses, we excluded patient samples with quality control warnings (*n* = 7) in the biomarker assays in either BL or FU2 measurements.

Moreover, the effects of VIDS on absolute IL-6 serum concentrations were further evaluated which exhibited statistically significant relative concentration changes following VIDS. A total of 11 samples (five from placebo and six from intervention) were excluded from the Olink Flex absolute measurements because they had quality control warnings. We assessed the correlation between absolute and relative IL-6 using Pearson correlation coefficients. In addition to the linear regression estimations, the effect of VIDS on absolute IL-6 serum concentrations was also assessed by comparing the proportion of patients with low or high IL-6 between the placebo and vitamin D groups based on the previously reported clinical cut-off value of 7 pg/mL [15].

For the exploratory analyses, a total of 69 biomarkers were available after excluding 20 biomarkers with high proportion (≥25%) of values below the LOD (see the list of all excluded biomarkers in Supplementary Table 2). The ITT approach was applied to obtain univariable linear regression β-coefficients and their respective unadjusted *p* values.

Multiple imputation of missing values (covariates only) was conducted using the MICE package in R statistical software. All statistical tests were performed using R-statistical software (version 4.3), and two-sided test significance levels were set at *p* values < 0.05.

## Results

### Patient characteristics at baseline

Patient characteristics at recruitment are presented in Table 1. The age distribution of included patients was similar in the placebo and VIDS groups, with a median age of 61 years (interquartile range [IQR] 56–68) and 60 years (IQR 55–69), respectively. In both arms, there were more male than female patients, presumably reflecting the higher incidence of CRC in males. More patients were diagnosed with stage IV in the intervention than in the placebo group (10% vs. 4.6%, respectively), whereas a higher proportion of patients received radiotherapy in the placebo compared to the intervention group (31% vs. 13%).

**Table 1:** Baseline characteristics of included participants.

Baseline characteristics at recruitment	Placebo, *n* = 65^a^	Treatment, *n* = 61^a^
Age [Median; IQR]	61 (56, 68)	60 (55, 69)
Sex female	17 (26%)	22 (36%)
Male	48 (74%)	39 (64%)
CRC stage at diagnosis		
I	19 (29%)	17 (28%)
II	19 (26%)	23 (35%)
III	24 (34%)	15 (23%)
IV	3 (4.6%)	6 (10%)
Time since surgery		
0–1 month	3 (4.7%)	5 (8.3%)
1–3 months	23 (36%)	25 (42%)
3–6 months	8 (13%)	8 (13%)
6–9 months	10 (16%)	14 (23%)
9–12 months	13 (20%)	5 (8.3%)
>12 months	7 (11%)	3 (5.0%)
Previous chemotherapy	35 (54%)	33 (54%)
Previous radiotherapy	20 (31%)	8 (13%)
Diabetes	10 (16%)	12 (20%)
History of CVD^b^	0 (0%)	3 (4.9%)
Hypertension	35 (57%)	31 (51%)
Body Mass Index (kg/m^2^) [Median; IQR]	27.2 (24.0, 29.4)	26.5 (24.5, 29.5)
<25	22 (34%)	19 (31%)
25−<30	27 (42%)	29 (48%)
≥30	16 (25%)	13 (21%)
Alcohol consumption^c^	59 (94%)	55 (92%)
Smoking status		
Never	24 (39%)	26 (43%)
Former	34 (55%)	24 (39%)
Current	4 (6%)	11 (18%)
Physical activity^d^		
Low	33 (54%)	28 (46%)
Adequate	8 (46%)	33 (54%)
25(OH)D nmol/l [Median; IQR]	20 (12, 28)	24 (15, 35)

Missing: CRC Stage at Diagnosis (*n* = 1), Time Since Surgery (*n* = 2), Previous Radiotherapy (*n* =  1), Diabetes (*n* = 3), History of CVD (*n* = 4), Hypertension (*n* = 4), Alcohol Consumption (*n* = 3), Smoking Status (*n* = 3), Physical Activity (*n* = 4).

*25 (OH)D* 25-hydroxyvitamin D, *CRC* colorectal cancer, *CVD* cardiovascular disease, *IFN-γ* interferon gamma, *IL-6* interleukin 6, *IQR* interquartile range, *MMP-1* matrix metalloproteinase-1.

^a^n (%) unless otherwise stated.

^b^CVD was defined as having diagnosed of Myocardial Infarction, Stroke, or Congestive Heart Failure.

^c^During the year before the CRC diagnosis.

^d^During the year before the CRC diagnosis; physical activity was assessed with the Rapid Assessment of Physical Activity questionnaire. However, we used the definition of the Healthy Lifestyle Score for healthy physical activity, which was as follows: at least 150 min of moderate-intensity or 75 min of vigorous-intensity aerobic physical activity throughout the week, or an equivalent combination of moderate-intensity physical activity is needed to meet the recommendations of healthy physical activity.

### Serum 25(OH)D concentrations and prevalence of serum vitamin D inadequacy

**Fig. 2:**
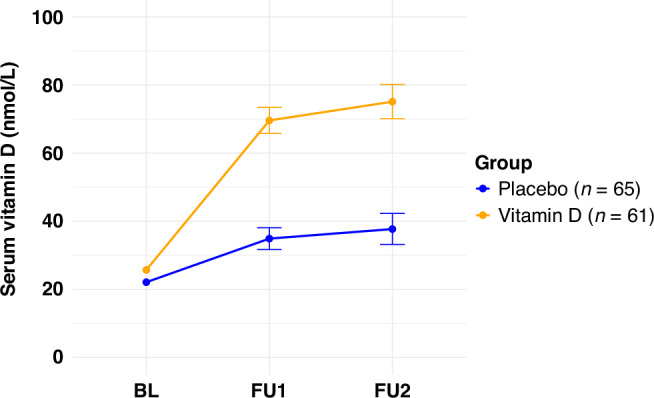
Change in mean serum vitamin D concentrations at different follow-up times. Mean serum vitamin D concentrations (nmol/L) at baseline (BL), first follow-up (FU1), and second follow-up (FU2) are shown for participants receiving placebo (*n* = 65) or vitamin D3 (*n* = 61). Data points represent means and error bars indicate standard errors of the mean.

The changes in mean serum 25(OH)D levels from BL to FU2 are graphically presented in Fig. 2 and tabulated in Supplementary Table 3. A much stronger increase in the mean serum 25(OH)D from BL to FU1 was observed in the intervention compared to the placebo group. At FU2, the prevalence of serum vitamin D inadequacy was significantly lower in the intervention group (Supplementary Table 4). In the PP analysis, serum 25(OH)D concentrations and prevalence of serum vitamin D inadequacy at different follow-up times were comparable to those observed in the ITT.

### Changes in inflammatory biomarker serum levels

**Fig. 3:**
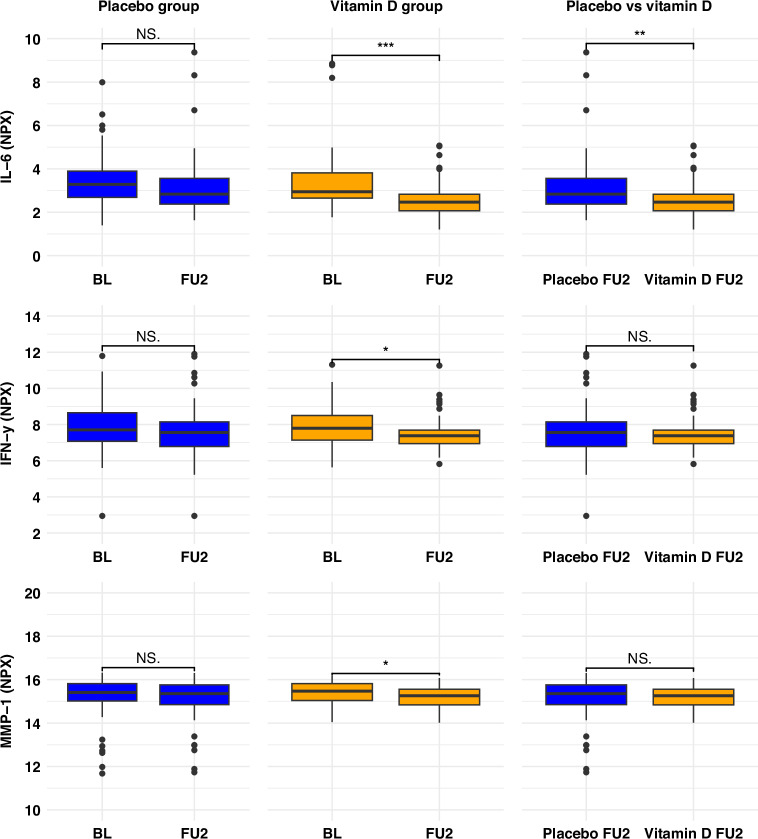
Differences in mean biomarker levels at baseline and end of trial (intention-to-treat analysis). Box-and-whisker plots show concentrations of interleukin-6 (IL-6), interferon-γ (IFN-γ), and matrix metalloproteinase-1 (MMP-1) at baseline (BL) and end of trial (FU2) in the placebo and vitamin D3 groups, and between-group differences at FU2. Boxes represent medians and interquartile ranges, whiskers indicate 1.5× interquartile range, and points denote outliers. Statistical significance is indicated as NS (not significant), *p<0.05, **p<0.01 and ***p<0.001.

The changes in within-study-arm mean NPX serum levels of IL-6, IFN-γ, and MMP-1 in the placebo and intervention groups at BL and FU2 are depicted in Fig. 3 with further details in Table 2. In general, all three biomarkers were decreased at FU2 compared to BL levels for both trial arms, although these changes were only statistically significant for IL-6 and IFN-γ in the VIDS group. Moreover, compared to the placebo group at FU2, IL-6 was significantly lower in the intervention group. However, these differences were not statistically significant for IFN-γ and MMP-1. In the PP analysis, results remained similar to those reported in the ITT analysis (Supplementary Table 5).

**Table 2:** Changes in serum biomarker levels: intention-to-treat analysis.

		Biomarker (NPX mean values)		
		IL-6	INF-γ	MMP-1
**Placebo** (*n* = 65)	BL	3.45 (1.25)	7.96 (1.35)	15.24 (0.96)
	FU2	3.18 (1.40)	7.63 (1.44)	15.11 (0.95)
	Within-study-arm mean difference (95% CI)	−0.22 (−0.61, 0.18)	−0.23 (−0.56, 0.10)	−0.10 (−0.18, −0.01)
	Percentage actual change from BL to FU2 (95% CI)^1^	−14.1 (−34.5, 13.3)	−14.7 (−32.2, 7.2)	−6.7 (−11.7, −0.69)
	*p* value^a^	0.276	0.166	0.031
**Intervention** (*n* = 61)	BL	3.47 (1.26)	7.96 (1.32)	15.36 (0.59)
	FU2	2.59 (0.86)	7.48 (0.94)	15.19 (0.50)
	Within-study-arm mean difference (95% CI)	**−0.80 (−1.16, −0.45)**	**−0.40 (−0.72, −0.09)**	**−0.18 (−0.27, −0.10)**
	Percentage actual change from BL to FU2 (95% CI)^1^	**−42.6 (−55.2, −26.8)**	**−24.2 (−39.3, −6.0)**	**−11.7 (−17.1, −6.7)**
	*p* value^a^	<0.001	0.013	<0.001
End of trial predicted changes (Intervention vs. Placebo group)^b^				
Model 1 (Unadjusted)	β−coefficient (95% CI)	**−0.59 (−1.01, −0.18)**	−0.15 (−0.59, 0.28)	0.09 (−0.19, 0.36)
	Percentage actual change from BL to FU2 (95% CI)^**2**^	**−33.6 (−50.3, −11.7)**	−9.9 (−33.6, 21.4)	6.4 (−12.3, 28.3)
	p−value	0.005	0.479	0.535
Model 2 (Adjusted)	β−coefficient (95% CI)	**−0.72 (−1.15, −0.29)**	−0.10 (−0.52, 0.35)	−0.08 (−0.20, 0.05)
	Percentage actual change from BL to FU2 (95% CI)^**2**^	**−39.3 (−54.9, −18.2)**	−6.7 (−30.3, 27.5)	−5.4 (−12.9, 3.5)
	*p* value	0.001	0.692	0.227

Serum biomarker values are in NPX values. For baseline and follow−up biomarker concentrations, mean values are presented with their respective standard deviations in parentheses. Mean changes are presented with their respective 95% confidence intervals. Bold figures are statistically significant after adjustment for family-wise error rate (FWER) using Bonferroni correction with an α-threshold of 0.0166.

*BL* baseline, *CI* confidence interval, *FU2* end of trial, *IFN-γ* interferon−gamma, *IL-6* interleukin-6, *MMP-1* matrix metalloproteinase-1, *NPX* normalized protein expression.

^a^*p* values based on paired *t*-tests.

^b^Based on linear regression models estimating biomarker changes in the intervention group versus placebo group (reference); Unadjusted biomarker changes were based on univariable model 1; Adjusted biomarker changes were based on multivariable model 2 controlled for baseline concentration of the respective inflammatory biomarker (continuous), baseline age (continuous), sex, baseline serum 25(OH)D (continuous), BMI (continuous), cancer stage (I, II, III, or IV), time since surgery (No surgery, 0−1, 2−3, 4−6, 7−9, 10−12, >12 months), previous chemotherapy and previous radiotherapy.

^1^Calculated from the formula (2^Mean NPX Difference^ − 1) × 100%.

^2^Calculated from the formula (2^β^ − 1) × 100%.

The results of the linear regression ITT analysis for estimating the changes in NPX serum concentrations of IL-6, IFN−γ, and MMP-1 due to VIDS are presented in Table 2. The adjusted estimated percentage actual change for IL-6 in the intervention compared to the placebo group was −39.3% (95% CI, −54.9 to −18.2%, *p* value = 0.001). However, for IFN-γ and MMP-1 these changes were not statistically significant [−6.7%; (95% CI, −30.3 to 27.5%) and −5.4%; (95% CI, −12.9 to 3.5%), respectively]. No violation of linear regression assumptions was observed (Supplementary Figs. 1–3). In the PP and sensitivity analyses, similar results were observed as for the ITT analysis, although with a slightly more pronounced effect of VIDS on IL−6 (Supplementary Tables 6 and 7).

Absolute and relative IL-6 were highly correlated (*r* > 0.8, *p* < 0.001) (Supplementary Fig. 4). The effects of VIDS on absolute IL-6 concentrations were further evaluated to assess the robustness of our promising results obtained with the relative concentration quantification. The within-arm median change in IL-6 at the end-of-trial was much higher in the VIDS group compared to the placebo group (−39.37% vs. −24.20%) (Supplementary Table 8). The estimated change in absolute IL-6 at the end-of-trial for the intervention compared to the placebo group was −46.8% (95% CI, −62.4 to −24.2%, *p* < 0.001) (Supplementary Table 9). No violation of linear regression assumptions was observed (Supplementary Fig. 5). By categorizing patients into low and high IL-6, the VIDS group showed a significantly lower proportion of patients with elevated IL-6 at the end of trial compared to the placebo group (10.9% vs. 28.3%, *p* = 0.019) (Supplementary Table 10).

In the exploratory analyses (Supplementary Table 11), VIDS significantly reduced CUB domain-containing protein-1 (CDCP-1), C-X-C motif chemokine (CXCL)-11, and CXCL-6 compared to placebo (unadjusted estimated change: −11.1%, *p* = 0.03; −17.1%, *p* = 0.04; and −13.5%, *p* = 0.02, respectively).

## Discussion

### Summary of findings

We assessed the impact of personalized VIDS on inflammatory markers in 126 CRC patients who had low initial serum 25(OH)D levels (<60 nmol/L). Patients who received a tailored loading dose of VIDS, followed by 2000 IU/day of VIDS for 12 weeks exhibited substantial elevations in serum 25(OH)D and significant decreases in serum IL-6 levels compared to those in the placebo group.

### Interpretation of findings

Whereas in our current trial we observed a 39% reduction, a previous RCT showed about 15% reduction in IL-6 serum levels in the VIDS compared to placebo group among CRC patients [16]. A plausible explanation for the stronger effect seen in our study was that VIDS was tailored to patients with low serum 25(OH)D in our study, in contrast to the previous RCT. In a meta-analysis of RCTs including patients with cancer and precancerous lesions, VIDS showed reduced serum TNF-α, IL-6, and CRP compared to the control group, although the effects were only statistically significant for TNF-α [8]. However, the RCTs included in this previous meta-analysis had several methodological limitations, including the application of uniform VIDS doses without accounting for critical factors such as initial vitamin D status, BMI, and the specific supplementation regimen (bolus vs. daily) [17]. Notably, supplementation appears to be most beneficial for individuals deficient in vitamin D, and there is a pronounced sequestration of 25(OH)D in individuals with obesity compared to people in the normal weight range [18]. Further, emerging data suggest superior outcomes with low-dose intermittent regimens of VIDS compared to high-dose bolus regimens in ameliorating vitamin D deficiency [19].

Calcitriol, the most biologically active metabolite of vitamin D, exerts its effects via the VDR, which regulates vitamin D-responsive gene expression across a variety of human cell types [20]. Calcitriol is a potent hormone that influences the transcription of more than 200 genes, thereby directly or indirectly affecting cellular processes, such as immune responses [21]. Specifically, calcitriol is known to suppress the activity of the nuclear factor ‘kappa-light-chain-enhancer’ of activated B-cells, a key regulator of inflammation, and can also mitigate immune-cell-mediated inflammatory responses [20]. Consequently, VIDS holds potential clinical value in attenuating inflammation-driven tumor progression for cancers such as CRC, where inflammatory cytokines like IL-6, TNF-α, and CRP are prominently elevated [21].

IL-6 is a principal pro-inflammatory cytokine positively associated with neoplastic proliferation, higher tumor grade, and high mortality rates in CRC patients [22]. The pro-tumorigenic role of IL-6 is mediated through the Janus Kinase/Signal Transducer and Activator of Transcription 3 (JAK/STAT3) signaling pathway. Thus, targeting the IL-6/JAK/STAT3 signaling axis has emerged as a viable therapeutic approach in CRC management, offering potential for directly suppressing cancer cell proliferation and enhancing antitumor immunity [23]. Specifically, the FDA-approved humanized monoclonal anti-IL-6R antibody Tocilizumab has been shown to disrupt JAK/STAT3 pathway activation, thereby augmenting the efficacy of chemotherapeutic agents [12]. VIDS could be an alternative to Tocilizumab because it has much less adverse events. By reducing IL-6 levels, it could play a critical role in modulating both inflammation and tumor progression [4], and thereby potentially enhance the health-related quality of life (HRQoL).

Mechanistic research proposes that calcitriol could modulate immune responses in CRC by repressing IFN-γ gene transcription in T cells, thereby diminishing IFN-γ production [24]. Furthermore, in vitro experiments have shown that vitamin D can reduce IFN-γ output by peripheral blood mononuclear cells [25]. Despite these connections, personalized VIDS showed only a small and non-significant impact on IFN-γ levels in our patient cohort. Evidence from multiple studies has established that MMP-1 is elevated in CRC tissue and correlates with poorer prognosis and increased metastatic risk [26–28]. Although the influence of VIDS on MMP-1 in CRC remains unexplored, studies in other contexts, such as uterine fibroids, indicate that calcitriol can downregulate the expression and activity of specific MMPs, including MMP-2 and MMP-9 [29]. Finally, our exploratory analysis revealed potential reductions in CDCP1, CXCL11, and CXCL6 due to VIDS. However, no research has yet specifically investigated the effects of VIDS on these markers in CRC, highlighting the need for further studies in this regard.

### Implications and future research

Routine screening and correction of vitamin D inadequacy in clinical settings could be beneficial for CRC patients, given the associations between low vitamin D levels, chronic inflammation, and adverse clinical outcomes. In addition to the pleiotropic benefits of vitamin D, including bone and muscle health, patients with CRC might derive significant benefits from the anti-inflammatory properties of VIDS as a supportive therapy post-treatment. Moreover, compared to e.g., anti-IL-6 biologicals, VIDS presents a potentially cost-effective option, considering its safety profile [14, 30], affordability, and wide availability.

### Strengths and limitations

Strengths include the careful selection of CRC patients with low serum 25(OH)D levels and the administration of personalized VIDS. We carefully selected inflammatory markers known to be prognostic indicators for CRC patients, enhancing the relevance and utility of our findings. Nevertheless, the power for detecting potential smaller effects on inflammatory biomarkers other than IL-6 was still limited.

## Conclusion

Personalized VIDS in CRC patients with low vitamin D status significantly reduced serum IL-6 levels. VIDS could be useful for inflammation management in CRC patients, potentially improving long-term prognosis and HRQoL. Future RCTs should assess the clinical significance of IL-6 modulation by VIDS.

## Supplementary information

Effects of personalized vitamin D3 on inflammation in colorectal cancer patients: a randomized trial

## Data Availability

Deidentified individual participant data, statistical code and any other materials can be requested from the corresponding author (see more details on https://inrepo02.dkfz.de/record/157452).

## Code availability

Deidentified individual participant data, statistical code and any other materials can be requested from the corresponding author (see more details on https://inrepo02.dkfz.de/record/157452).
