# Supplementary material for: Effects of personalized vitamin D3 on inflammation in colorectal cancer patients: a randomized trial
Source: Br J Cancer. 2026 Jan 8;134(6):874–80. doi: 10.1038/s41416-025-03333-6 (PMC12960710; doi:10.1038/s41416-025-03333-6)
Supplement: Supplementary file 1 — Effects of personalized vitamin D3 on inflammation in colorectal cancer patients: a randomized trial [file 41416_2025_3333_MOESM1_ESM.docx]

**SUPPLEMENTARY MATERIALS**

**Contents**

[**Supplementary Methods.** 2](#_Toc211260373)

[**Supp. Fig 1.** Test for linear regression assumptions for estimating IL-6 change in the ITT analysis. 4](#_Toc211260374)

[**Supp. Fig 2.** Test for linear regression assumptions for estimating IFN-gamma change in the ITT analysis 5](#_Toc211260375)

[**Supp. Fig 3.** Test for linear regression assumptions for estimating MMP-1 change in the ITT analysis. 6](#_Toc211260376)

[**Supp. Fig 4.** Correlation between absolute and relative IL-6 at baseline and end-of-trial. 7](#_Toc211260377)

[**Supp. Fig 5.** Test for linear regression assumptions for estimating IL-6 change. 8](#_Toc211260378)

[**Supp. Table 1.** List of biomarkers measured with Olink Target 96 Inflammation Panel kits. 9](#_Toc211260379)

[**Supp. Table 2**. Biomarkers excluded due to high proportion (≥25%) of values below the Limit of Detection at baseline. 11](#_Toc211260380)

[**Supp. Table 3.** Serum 25(OH)D concentration at different follow-up time-points. 12](#_Toc211260381)

[**Supp. Table 4**. Prevalence of vitamin D inadequacy [25(OH)D levels < 50 nmol/L] at different follow-up times. 13](#_Toc211260382)

[**Supp. Table 5**. Differences in mean biomarker levels between placebo and intervention groups at the end of trial (Per Protocol, n = 120) 14](#_Toc211260383)

[**Supp. Table 6.** Linear regression estimates of the change in inflammatory biomarker levels due to vitamin D3 supplementation at the end of trial (Per Protocol, n = 120) 15](#_Toc211260384)

[**Supp. Table 7.** Sensitivity analysis: Linear regression estimates of the change in inflammatory biomarker levels due to vitamin D3 supplementation at the end of trial excluding patient samples with Quality Control Warnings (Per Protocol, n = 113) 16](#_Toc211260385)

[**Supp. Table 8.** Differences in median biomarker levels between placebo and intervention groups at the end of trial (n = 115). 17](#_Toc211260386)

[**Supp. Table 9.** Linear regression estimates of the change in inflammatory biomarker levels due to vitamin D3 supplementation at the end of trial (n = 115). 18](#_Toc211260387)

[**Supp. Table 10.** Comparison of Interleukin-6 status by treatment group at baseline and end-of-trial 19](#_Toc211260388)

[**Supp. Table 11.** Exploratory linear regression estimates of the effects of vitamin D3 supplementation on inflammatory biomarkers at the end of trial (Intention-To-Treat Analysis, n=126). 20](#_Toc211260389)

**Supplementary Methods.**

*Study Design and Participants*

Briefly, in this ongoing multicenter, parallel-group, randomized, double-blind, placebo-controlled clinical trial, CRC patients (≥18 years of age) are recruited from five German rehabilitation clinics. Study inclusion requires a diagnosis of CRC and its treatment in the last 12 months (surgical removal of the tumor, chemotherapy or radiotherapy) and at least three weeks in-patient rehabilitation in a cooperating clinic. The most important exclusion criteria include 25(OH)D) levels ≥ 60 nmol/L, BMI > 40kg/m^2^, high-dose VIDS therapy (≥ 2000 IU daily or similar dosage), high-dose calcium therapy (> 1000 mg calcium daily), hypercalcemia, hypercalciuria, and severe renal impairment (eGFR < 30 ml/min/1,73m^2^).

*Inflammatory Biomarker Measurements*

Serum samples were analyzed in a first run of available samples at the end of 2022 in the Research Unit Protein Science, German Research Center for Environmental Health in Munich. A second measurement round was conducted in the Genomics and Proteomics Core Facility at the German Cancer Research Center in Heidelberg at the end of 2023. Sixteen bridging samples that had passed quality control checks and exhibited high detectability from the two time points (BL and FU2) were selected for the normalization process. We used the *link_normalization_bridge()* function within the OlinkAnalyze R package (version 4.1.2) specifically designed for bridging normalization and maintained by the Olink Proteomics Data Science Team. Biomarkers with ≥ 25% of the values below the lower limit of detection (LOD) at BL were excluded from the analyses (n = 20) (see **Supp. Table 2**), while for biomarkers with less than 25% of values below the LOD (n = 11), these values were replaced by LOD/√2. Overall, 72 out of the 92 biomarkers were evaluated.

*Multiple imputation*

Five imputation datasets with 30 iterations were applied using the following imputation model including all assessed variables that theoretically predict inflammatory response: Treatment arm (dichotomous: placebo or vitamin D3 supplementation), baseline age (continuous), sex (dichotomous), school education (≤ 9, 10-11, ≥ 12 years), serum 25(OH)D level (continuous), cancer stage (I, II, III, or IV), time since diagnosis (continuous), time since CRC surgery (No surgery, 0-1, 2-3, 4-6, 7-9, 10-12, >12 months), time since last chemotherapy (No chemotherapy, 0-1, 2-3, 4-6, 7-9, 10-12, >12 months), time since last radiotherapy (No radiotherapy, 0-1, 2-3, 4-6, 7-9, 10-12, >12 months), planned chemotherapy or radiotherapy in next 3 months (No, yes chemotherapy, yes radiotherapy, yes both), stoma at baseline (dichotomous), subjective pain burden (continuous scale from 0-5), subjective exhaustion burden (continuous scale from 0-5), diabetes at baseline (dichotomous), cardiovascular disease at baseline (dichotomous, defined by coronary heart disease, history of myocardial infarction or revascularization of coronary arteries), heart failure at baseline (dichotomous), history of stroke (dichotomous), chronic obstructive pulmonary disease at baseline (dichotomous), asthma at baseline (dichotomous), renal failure at baseline (dichotomous), arthropathy at baseline (dichotomous, defined as arthritis, arthrosis or other rheumatic joint disease), diarrhoea in last week (none, a little, moderate, a lot), baseline BMI (continuous), baseline smoking status (never, former, current), baseline alcohol consumption (none, low, moderate, high), baseline physical activity meeting WHO recommendation (dichotomous), baseline red meat consumption (never, up to 3 times a month, 1-3 days a week, 4-6 days per week, daily), frailty (non-frail, pre-frail, frail), baseline global quality of life (continuous scale from 1-7), baseline and 12-week OLINK inflammation panel biomarker levels of all biomarkers meeting inclusion criteria (continuous), and baseline and 12-week follow-up quality control warning for blood samples (declared as “Sample Failed” in the OLINK analysis report).

**Supp. Fig 1.** Test for linear regression assumptions for estimating IL-6 change in the ITT analysis.

| **Linearity^1^**  **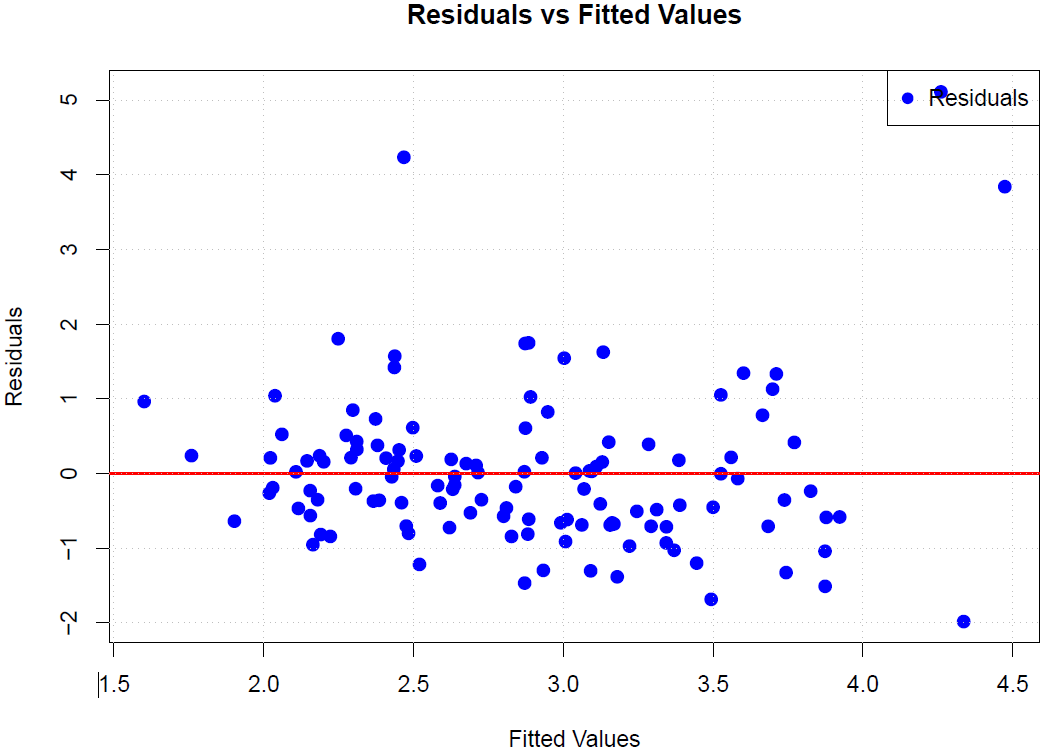** | **Normality of Residuals**  (Shapiro-Will test = 0.86, p < 0.001)**^2^**  **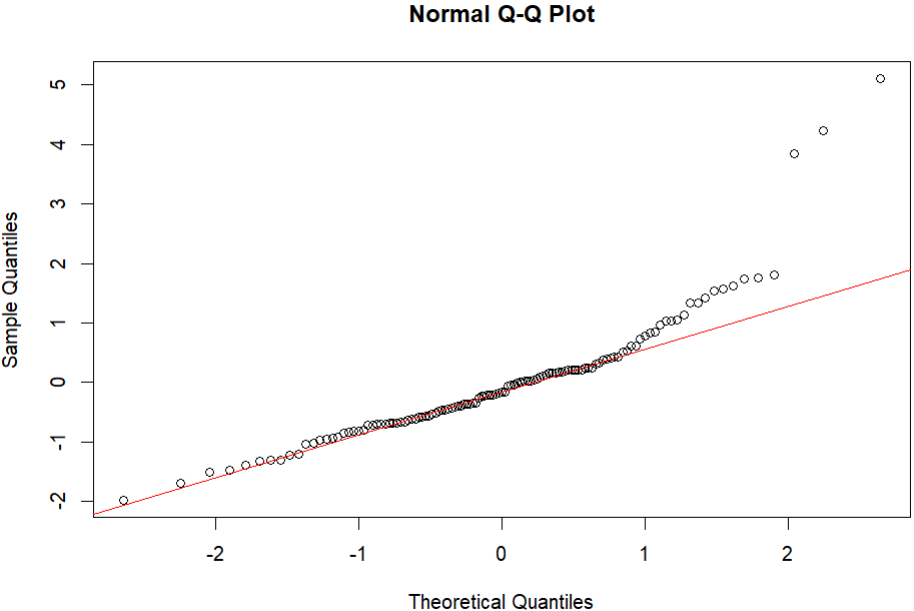** |
| --- | --- |
| **Homoscedasticity^3^**  Breusch-Pagan test = 19.59; df = 13; p = 0.11 | **Multicollinearity^4^**  Variance Inflation Factors (VIF) range from 1.13 – 2.85 |

^1^Linearity Visual Inspection: Plot residuals vs. fitted values. If the plot shows a random pattern, linearity is likely satisfied.

^2^Normality of Residuals: Residuals should be approximately normally distributed. Q-Q Plot: Compare the distribution of residuals to a normal distribution. Shapiro-Wilk Test: Formal test for normality. The Shapiro-Wilk test is used to determine whether a sample comes from a normally distributed population. H₀: The data is normally distributed. H₁: The data is not normally distributed.

^3^Breusch-Pagan Statistic: quantifies the degree of heteroscedasticity detected in the residuals. H₀: Homoscedasticity (the residuals have constant variance). H₁: Heteroscedasticity (the residuals do not have constant variance).

^4^Variance Inflation Factor (VIF) is a measure of how much the variance of a regression coefficient is inflated due to multicollinearity among the predictor variables. VIF = 1: There is no multicollinearity between the predictor variable and the other predictor variables in the model. 1 < VIF < 5: Moderate multicollinearity exists, but it is generally not severe enough to require corrective measures. VIF ≥ 5: High multicollinearity is present. This may indicate that the predictor variable is highly collinear with other predictor variables, which can lead to unreliable estimates of regression coefficients. VIF ≥ 10: Very high multicollinearity exists, often considered a sign that the model has severe multicollinearity issues, and corrective measures are typically required.

**Supp. Fig 2.** Test for linear regression assumptions for estimating IFN-gamma change in the ITT analysis

| **Linearity^1^**  **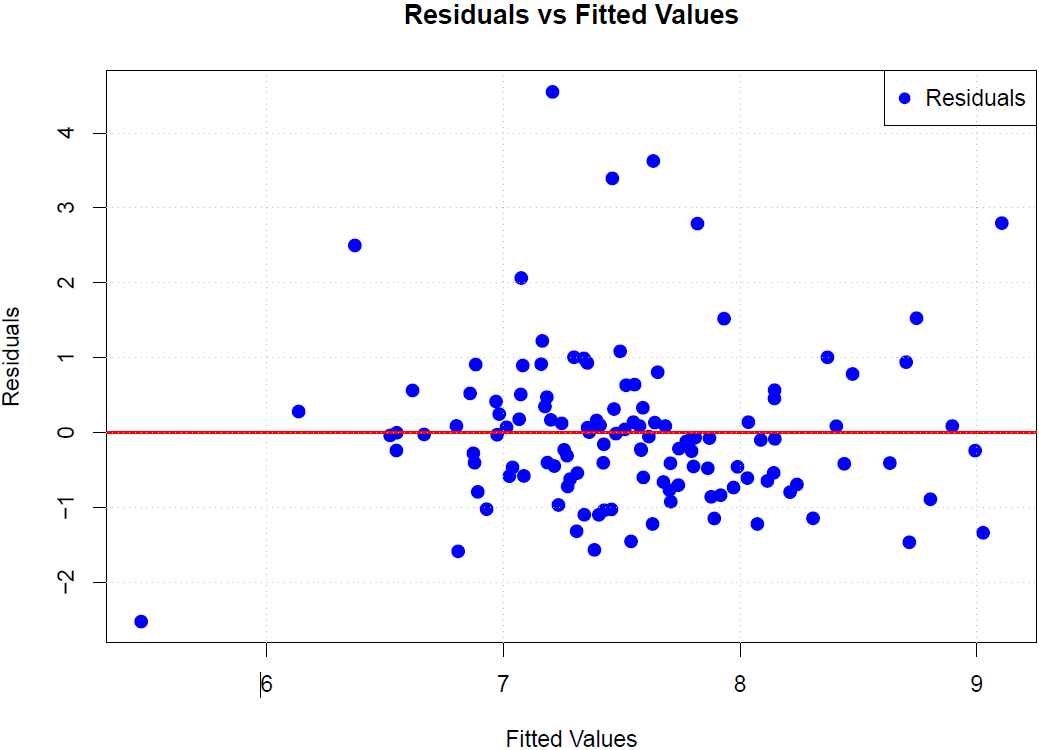** | **Normality of Residuals**  (Shapiro-Will test = 0.89, p < 0.001)**^2^**  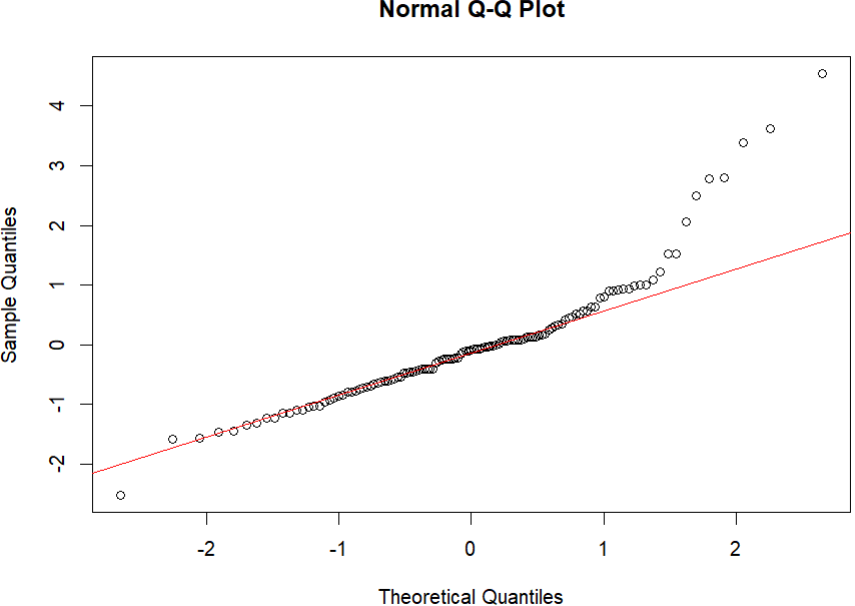 |
| --- | --- |
| **Homoscedasticity^3^**  Breusch-Pagan test = 7.36; df = 13; p-value = 0.88 | **Multicollinearity^4^**  Variance Inflation Factors (VIF) range from 1.08 – 2.91 |

^1^Linearity Visual Inspection: Plot residuals vs. fitted values. If the plot shows a random pattern, linearity is likely satisfied.

^2^Normality of Residuals: Residuals should be approximately normally distributed. Q-Q Plot: Compare the distribution of residuals to a normal distribution. Shapiro-Wilk Test: Formal test for normality. The Shapiro-Wilk test is used to determine whether a sample comes from a normally distributed population. H₀: The data is normally distributed. H₁: The data is not normally distributed.

^3^Breusch-Pagan Statistic: quantifies the degree of heteroscedasticity detected in the residuals. H₀: Homoscedasticity (the residuals have constant variance). H₁: Heteroscedasticity (the residuals do not have constant variance).

^4^Variance Inflation Factor (VIF) is a measure of how much the variance of a regression coefficient is inflated due to multicollinearity among the predictor variables. VIF = 1: There is no multicollinearity between the predictor variable and the other predictor variables in the model. 1 < VIF < 5: Moderate multicollinearity exists, but it is generally not severe enough to require corrective measures. VIF ≥ 5: High multicollinearity is present. This may indicate that the predictor variable is highly collinear with other predictor variables, which can lead to unreliable estimates of regression coefficients. VIF ≥ 10: Very high multicollinearity exists, often considered a sign that the model has severe multicollinearity issues, and corrective measures are typically required.

**Supp. Fig 3.** Test for linear regression assumptions for estimating MMP-1 change in the ITT analysis.

| **Linearity^1^**  **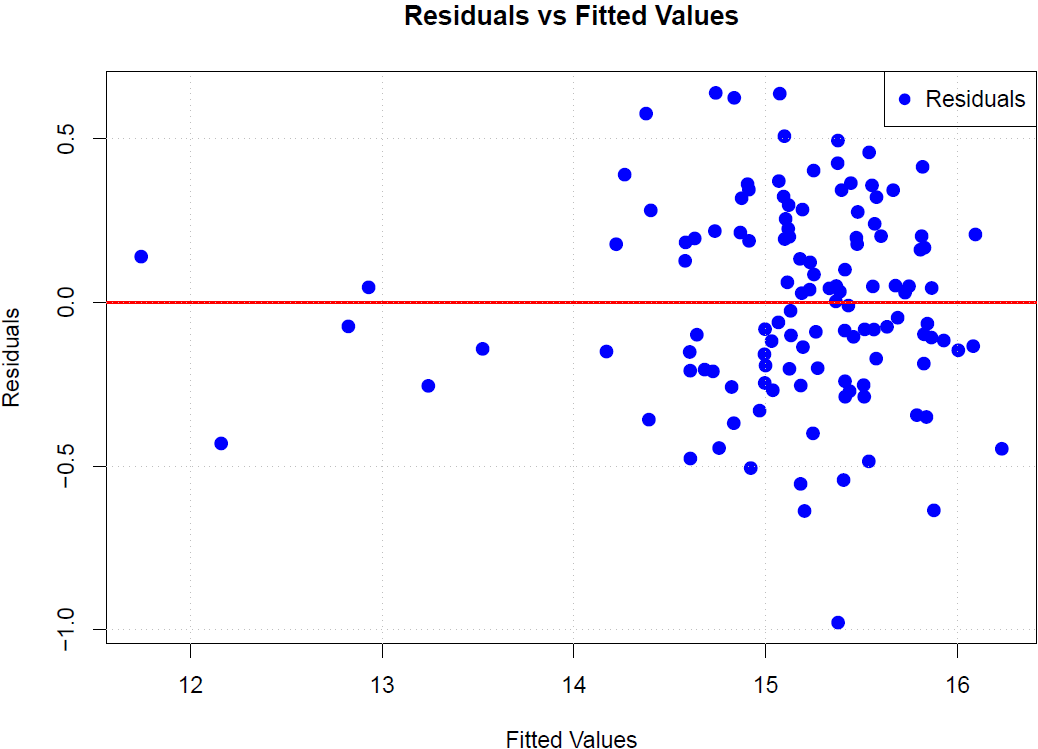** | **Normality of Residuals**  (Shapiro-Will test = 0.99, p = 0.56)**^2^**  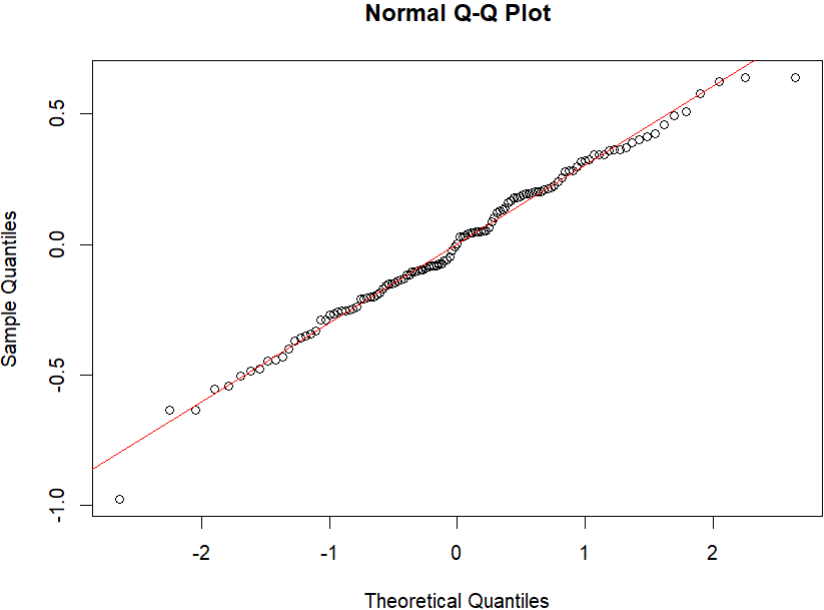 |
| --- | --- |
| **Homoscedasticity^3^**  Breusch-Pagan test = 15.73; df = 13; p-value = 0.26 | **Multicollinearity^4^**  Variance Inflation Factors (VIF) range from 1.08 – 2.83 |

^1^Linearity Visual Inspection: Plot residuals vs. fitted values. If the plot shows a random pattern, linearity is likely satisfied.

^2^Normality of Residuals: Residuals should be approximately normally distributed. Q-Q Plot: Compare the distribution of residuals to a normal distribution. Shapiro-Wilk Test: Formal test for normality. The Shapiro-Wilk test is used to determine whether a sample comes from a normally distributed population. H₀: The data is normally distributed. H₁: The data is not normally distributed.

^3^Breusch-Pagan Statistic: quantifies the degree of heteroscedasticity detected in the residuals. H₀: Homoscedasticity (the residuals have constant variance). H₁: Heteroscedasticity (the residuals do not have constant variance).

^4^Variance Inflation Factor (VIF) is a measure of how much the variance of a regression coefficient is inflated due to multicollinearity among the predictor variables. VIF = 1: There is no multicollinearity between the predictor variable and the other predictor variables in the model. 1 < VIF < 5: Moderate multicollinearity exists, but it is generally not severe enough to require corrective measures. VIF ≥ 5: High multicollinearity is present. This may indicate that the predictor variable is highly collinear with other predictor variables, which can lead to unreliable estimates of regression coefficients. VIF ≥ 10: Very high multicollinearity exists, often considered a sign that the model has severe multicollinearity issues, and corrective measures are typically required.

**Supp. Fig 4.** Correlation between absolute and relative IL-6 at baseline and end-of-trial.

| 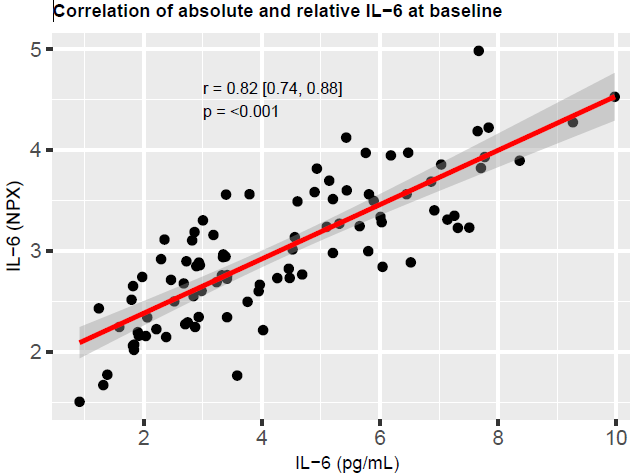 | 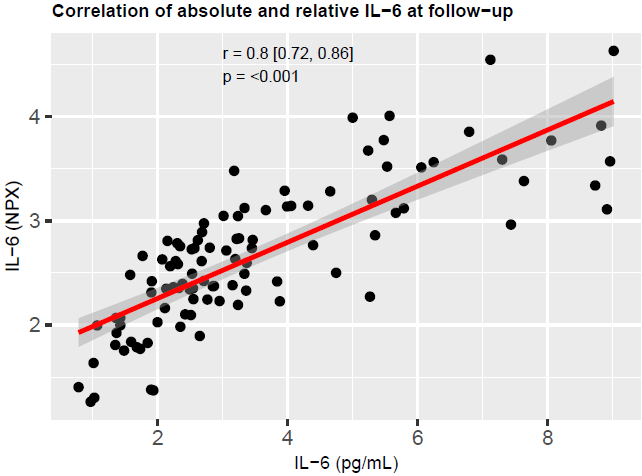 |
| --- | --- |

Abbreviations: IL-6, interleukin 6; NPX, normalized protein expression; r, Pearson correlation co-efficient.

**Supp. Fig 5.** Test for linear regression assumptions for estimating IL-6 change.

| Model 1 | Model 2 |
| --- | --- |
| 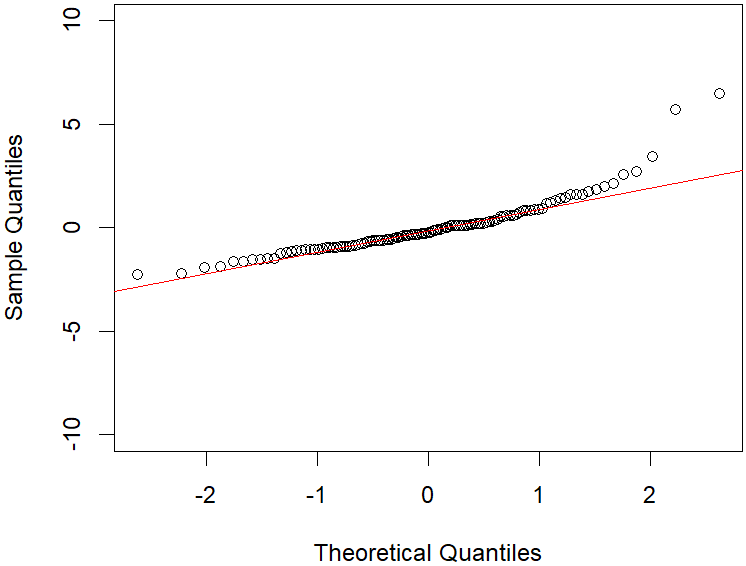 | 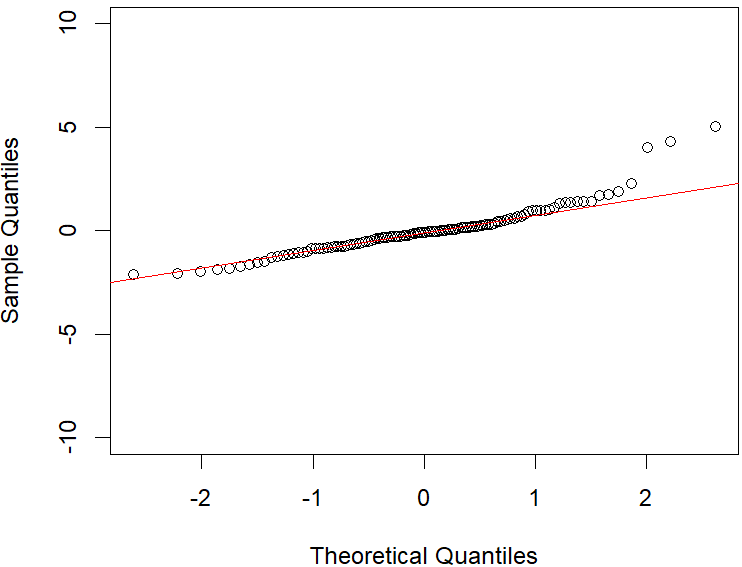 |

Notes: Model 1 is for the univariable linear regression, Model 2 is for the multivariable adjusted linear regression.

**Supp. Table 1.** List of biomarkers measured with Olink Target 96 Inflammation Panel kits.

| **Abbreviation** | **Biomarker name** |
| --- | --- |
| 4E-BP1 | Eukaryotic translation initiation factor 4E-binding protein 1 |
| ADA | Adenosine Deaminase |
| ARTN | Artemin |
| AXIN1 | Axin-1 |
| Beta-NGF | Beta-nerve growth factor |
| CASP-8 | Caspase-8 |
| CCL11 | Eotaxin |
| CCL19 | C-C motif chemokine 19 |
| CCL20 | C-C motif chemokine 20 |
| CCL23 | C-C motif chemokine 23 |
| CCL25 | C-C motif chemokine 25 |
| CCL28 | C-C motif chemokine 28 |
| CCL3 | C-C motif chemokine 3 |
| CCL4 | C-C motif chemokine 4 |
| CD244 | Natural killer cell receptor 2B4 |
| CD40 | CD40L receptor |
| CD5 | T-cell surface glycoprotein CD5 |
| CD6 | T cell surface glycoprotein CD6 isoform |
| CD8A | T-cell surface glycoprotein CD8 alpha chain |
| CDCP1 | CUB domain-containing protein 1 |
| CSF-1 | Macrophage colony-stimulating factor 1 |
| CST5 | Cystatin D |
| CX3CL1 | Fractalkine |
| CXCL1 | C-X-C motif chemokine 1 |
| CXCL10 | C-X-C motif chemokine 10 |
| CXCL11 | C-X-C motif chemokine 11 |
| CXCL5 | C-X-C motif chemokine 5 |
| CXCL6 | C-X-C motif chemokine 6 |
| CXCL9 | C-X-C motif chemokine 9 |
| DNER | Delta and Notch-like epidermal growth factor-related receptor |
| EN-RAGE | Protein S100-A12 |
| FGF-19 | Fibroblast growth factor 19 |
| FGF-21 | Fibroblast growth factor 21 |
| FGF-23 | Fibroblast growth factor 23 |
| FGF-5 | Fibroblast growth factor 5 |
| Flt3L | Fms-related tyrosine kinase 3 ligand |
| GDNF | Glial cell line-derived neurotrophic factor |
| HGF | Hepatocyte growth factor |
| IFN_gamma | Interferon gamma |
| IL1_alpha | Interleukin-1 alpha |
| IL-10 | Interleukin-10 |
| IL-10RA | Interleukin-10 receptor subunit alpha |
| IL-10RB | Interleukin-10 receptor subunit beta |
| IL-12B | Interleukin-12 subunit beta |
| IL13 | Interleukin-13 |
| IL-15RA | Interleukin-15 receptor subunit alpha |
| IL-17A | Interleukin-17A |
| IL-17C | Interleukin-17C |
| IL-18 | Interleukin-18 |
| IL-18R1 | Interleukin-18 receptor 1 |
| IL2 | Interleukin-2 |
| IL20 | Interleukin-20 |
| IL-20RA | Interleukin-20 receptor subunit alpha |
| IL22-RA1 | Interleukin-22 receptor subunit alpha-1 |
| IL24 | Interleukin-24 |
| IL2RB | Interleukin-2 receptor subunit beta |
| IL33 | Interleukin-33 |
| IL4 | Interleukin-4 |
| IL-5 | Interleukin-5 |
| IL-6 | Interleukin-6 |
| IL-7 | Interleukin-7 |
| IL-8 | Interleukin-8 |
| LAP TGF-beta-1 | Latency-associated peptide transforming growth factor beta-1 |
| LIF | Leukemia inhibitory factor |
| LIFR | Leukemia inhibitory factor receptor |
| MCP-1 | Monocyte chemotactic protein 1 |
| MCP-2 | Monocyte chemotactic protein 2 |
| MCP-3 | Monocyte chemotactic protein 3 |
| MCP-4 | Monocyte chemotactic protein 4 |
| MMP-1 | Matrix metalloproteinase-1 |
| MMP-10 | Matrix metalloproteinase-10 |
| NRTN | Neurturin |
| NT-3 | Neurotrophin-3 |
| OPG | Osteoprotegerin |
| OSM | Oncostatin-M |
| PD-L1 | Programmed cell death 1 ligand 1 |
| SCF | Stem cell factor |
| SIRT2 | SIR2-like protein 2 |
| SLAMF1 | Signaling lymphocytic activation molecule |
| ST1A1 | Sulfotransferase 1A1 |
| STAMBP | STAM-binding protein |
| TGF-alpha | Transforming growth factor alpha |
| TNF | Tumor necrosis factor |
| TNFB | TNF-beta |
| TNFRSF9 | Tumor necrosis factor receptor superfamily member 9 |
| TNFSF14 | Tumor necrosis factor ligand superfamily member 14 |
| TRAIL | TNF-related apoptosis-inducing ligand |
| TRANCE | TNF-related activation-induced cytokine |
| TSLP | Thymic stromal lymphopoietin |
| TWEAK | Tumor necrosis factor (Ligand) superfamily, member 12 |
| uPA | Urokinase-type plasminogen activator |
| VEGF-A | Vascular endothelial growth factor-A |

**Supp. Table 2**. Biomarkers excluded due to high proportion (≥25%) of values below the Limit of Detection at baseline.

| Abbreviation | Biomarker name | Proportion of Values < LOD |
| --- | --- | --- |
| ARTN | Artemin | 69.2% |
| Beta-NGF | Beta-nerve growth factor | 93.0% |
| CXCL-10 | C-X-C motif chemokine 10 | 30.4% |
| GDNF | Glial cell line-derived neurotrophic factor | 28.0% |
| IL-1 alpha | Interleukin-1 alpha | 83.6% |
| IL-17A | Interleukin-17A | 32.7% |
| IL-20 | Interleukin-20 | 47.7% |
| IL-20RA | Interleukin-20 receptor subunit alpha | 44.4% |
| IL-22 RA1 | Interleukin-22 receptor subunit alpha-1 | 55.1% |
| IL-24 | Interleukin-24 | 87.9% |
| IL-2RB | Interleukin-2 receptor subunit beta | 58.4% |
| IL-13 | Interleukin-13 | 68.7% |
| IL-2 | Interleukin-2 | 88.3% |
| IL-33 | Interleukin-33 | 81.8% |
| IL-4 | Interleukin-4 | 53.7% |
| IL-5 | Interleukin-5 | 48.6% |
| LIF | Leukemia inhibitory factor | 60.7% |
| NRTN | Neurturin | 42.5% |
| NT-3 | Neurotrophin-3 | 25.2% |
| TSLP | Thymic stromal lymphopoietin | 79.9% |

**Abbreviations:** LOD, lower limit of detection

**Supp. Table 3.** Serum 25(OH)D concentration at different follow-up time-points.

| **Timepoint** | **Intention-to-Treat** | | | |
| --- | --- | --- | --- | --- |
|  | Placebo (n = 65) | | Treatment (n = 61) | |
|  | Mean (95% CI) | Change (95% CI) | Mean (95% CI) | Change (95% CI) |
| BL | 22.1 | - | 25.7 | - |
| FU1 | 34.9 (31.7, 38.1) | 12.8 (9.1, 16.4)^1^ | 69.6 (65.8, 73.4) | 44.2 (39.5, 49.0)^1^ |
| FU2 | 37.7 (33.2, 42.3) | 2.6 (-0.9, 6.0)^2^ | 75.1 (70.1, 80.2) | 5.8 (1.0, 10.6)^2^ |
| Overall Change (95%CI) | - | 15.6 (10.5, 20.7)^3^ | - | 49.4 (43.3, 55.5)^3^ |
|  | **Per Protocol** | | | |
|  | Placebo (n = 63) | | Treatment (n = 57) | |
| BL | 22.4 | - | 24.5 | - |
| FU1 | 35.0 (31.7, 38.4) | 12.7 (8.9, 16.4)^1^ | 68.4 (64.7, 72.1) | 43.9 (39.1, 48.7)^1^ |
| FU2 | 37.3 (32.7, 42.0) | 2.3 (-1.3, 5.8)^2^ | 75.1 (69.6, 80.6) | 6.7 (1.6, 11.9)^2^ |
| Overall Change (95%CI) |  | 14.9 (9.7, 20.1)^3^ |  | 50.7 (44.2, 57.1)^3^ |

Notes: Serum 25(OH)D values are in nmol/L.

^1^Difference between FU1 and BL mean values.

^2^Difference between FU2 and FU1 mean values.

^3^Difference between FU2 and BL mean values.

Abbreviations: BL, baseline; CI, confidence interval; FU1, end of rehabilitation; FU2, end of trial

**Supp. Table 4**. Prevalence of vitamin D inadequacy [25(OH)D levels < 50 nmol/L] at different follow-up times.

| **Timepoint** | **Intention-to-Treat** | |
| --- | --- | --- |
|  | Placebo (n = 65) | Treatment (n = 61) |
|  | Proportion (95% CI) | Proportion (95% CI) |
| BL | 100.0 | 98.4 |
| FU1 | 87.5 (76.8 – 94.4) | 8.3 (2.8 – 18.4) |
| FU2 | 76.9 (64.8 – 86.5) | 8.2 (2.7 – 18.1) |
|  |  |  |
|  | **Per Protocol** | |
|  | Placebo (n = 63) | Treatment (n = 57) |
| BL | 100.0 | 100.0 |
| FU1 | 87.1 (76.1 – 94.3) | 9.1 (3.0 – 19.9) |
| FU2 | 79.0 (66.8 – 88.3) | 9.1 (3.0 – 19.9) |

Notes: Proportions are presented as percentages

Abbreviations: BL, baseline; CI, confidence interval; FU1, end of rehabilitation; FU2, end of trial

**Supp. Table 5**. Differences in mean biomarker levels between placebo and intervention groups at the end of trial (Per Protocol, n = 120)

| **Treatment Group** | **Biomarker** (Mean NPX Values) | | |
| --- | --- | --- | --- |
|  | **IL-6** | **INF-γ** | **MMP-1** |
| Placebo (n = 63) | 3.19 (1.43) | 7.66 (1.45) | 15.15 (0.86) |
| Intervention (n = 57) | 2.53 (0.82) | 7.49 (0.96) | 15.19 (0.51) |
| Mean NPX Difference (95%CI) | **-0.66 (-1.07, -0.24)** | -0.17 (-0.60, 0.28) | 0.04 (-0.21, 0.30) |
| Percentage Actual Change from BL to FU2 (95% CI)^1^ | **-35.0 (-52.0, -15.0)** | -11.0 (-34.0, 21.0) | 3.0 (-13.0, 23.0) |
| p-value**^‡^** | 0.003 | 0.522 | 0.367 |

**^‡^**p-values based on t-tests

**^1^**Calculated from the formula (2^Mean NPX Difference^ - 1) x 100%.

Abbreviations: BL, baseline; CI, confidence interval; FU2, end of trial; IFN-γ, interferon-gamma; IL-6, interleukin-6; MMP-1, matrix metalloproteinase-1.

Notes: Serum mean NPX values are presented with their respective standard deviations in parentheses. Bold figures are statistically significant after adjustment for family-wise error rate (FWER) using Bonferroni correction with α-threshold of 0.0166.

**Supp. Table 6.** Linear regression estimates of the change in inflammatory biomarker levels due to vitamin D3 supplementation at the end of trial (Per Protocol, n = 120)

| **Biomarker^1^** | **Model 1** | | | | **Model 2** | | | |
| --- | --- | --- | --- | --- | --- | --- | --- | --- |
|  | β-coefficient  (95% CI) | SE | P-value | % Change (95% CI)^2^ | β-coefficient  (95% CI) | SE | P-value | % Change (95% CI)^2^ |
| IL-6 | **-0.66 (-1.08, -0.23)** | 0.22 | **0.003** | **-36.7 (-52.7, -14.7)** | **-0.71 (-1.16, -0.26)** | 0.23 | **0.002** | **-38.9 (-55.2, -16.5)** |
| IFN-γ | -0.16 (-0.61, 0.29) | 0.23 | 0.476 | -10.5 (-34.5, 22.3) | -0.19 (-0.57, 0.33) | 0.23 | 0.602 | -12.3 (-32.6, 25.7) |
| MMP-1 | 0.05 (-0.21, 0.30) | 0.13 | 0.727 | 3.5 (-13.5, 23.1) | -0.08 (-0.21, 0.05) | 0.06 | 0.212 | -5.4 (-13.5, 3.5) |

**^1^**Biomarker concentrations are in NPX values; Bold figures are statistically significant after adjustment for type-1 error (FWER) using Bonferroni correction with α-threshold of 0.0166.

**^2^**Calculated from the formula (2^β^ - 1) x 100%

Model 1, univariable; Model 2, adjusted for baseline concentration of the respective inflammatory biomarker (continuous), baseline age (continuous), sex, baseline serum 25(OH)D (continuous), BMI (continuous), cancer stage (I, II, III, or IV), time since surgery (No surgery, 0-1, 2-3, 4-6, 7-9, 10-12, >12 months), previous chemotherapy and previous radiotherapy.

Abbreviations: CI, confidence interval; IFN-γ, interferon-gamma; IL-6, interleukin-6; MMP-1, matrix metalloproteinase-1; NPX, normalized protein expression; SE, standard error

**Supp. Table 7.** Sensitivity analysis: Linear regression estimates of the change in inflammatory biomarker levels due to vitamin D3 supplementation at the end of trial excluding patient samples with Quality Control Warnings (Per Protocol, n = 113)

| **Biomarker^1^** | **Model 1** | | | | **Model 2** | | | |
| --- | --- | --- | --- | --- | --- | --- | --- | --- |
|  | β-coefficient  (95% CI) | SE | P-value | % Change (95% CI)^2^ | β-coefficient  (95% CI) | SE | P-value | % Change (95% CI)^2^ |
| IL-6 | **-0.65 (-1.10, -0.19)** | 0.23 | **0.006** | **-36.3 (-53.3, -12.3)** | **-0.79 (-1.27, -0.31)** | 0.24 | **0.002** | **-42.2 (-58.5, -19.3)** |
| IFN-γ | -0.15 (-0.64, 0.33) | 0.24 | 0.525 | -9.9 (-35.8, 25.7) | -0.09 (-0.58, 0.40) | 0.25 | 0.722 | -6.0 (-33.1, 32.0) |
| MMP-1 | 0.07 (-0.21, 0.35) | 0.14 | 0.621 | 5.0 (-13.5, 27.5) | -0.06 (-0.20, 0.07) | 0.07 | 0.342 | -4.1 (-12.9, 5.0) |

**^1^**Biomarker concentrations are in NPX values; Bold figures are statistically significant after adjustment for type-1 error (FWER) using Bonferroni correction with α-threshold of 0.0166.

**^2^**Calculated from the formula (2^β^ - 1) x 100%

Model 1, univariable; Model 2, adjusted for baseline concentration of the respective inflammatory biomarker (continuous), baseline age (continuous), sex, baseline serum 25(OH)D (continuous), BMI (continuous), cancer stage (I, II, III, or IV), time since surgery (No surgery, 0-1, 2-3, 4-6, 7-9, 10-12, >12 months), previous chemotherapy and previous radiotherapy.

Abbreviations: CI, confidence interval; IFN-γ, interferon-gamma; IL-6, interleukin-6; MMP-1, matrix metalloproteinase-1; NPX, normalized protein expression; SE, standard error

**Supp. Table 8.** Differences in median biomarker levels between placebo and intervention groups at the end of trial (n = 115).

| **Treatment Group Details** | | **Median IL-6 (IQR)**, *pg/mL* |
| --- | --- | --- |
| **Placebo**  (n = 60) | BL | 5.17 (2.94 – 7.73) |
|  | FU2 | 3.92 (2.54 – 7.34) |
|  | Within-Study-Arm Median Difference | -1.25 |
|  | Percentage Median Change from BL to FU2 | -24.20% |
|  | p-value**^*^** | 0.032 |
| **Intervention**  (n = 55) | BL | 4.47 (2.78 – 7.18) |
|  | FU2 | 2.71 (1.96 – 3.75) |
|  | Within-Study-Arm Median Difference | -1.76 |
|  | Percentage Median Change from BL to FU2 | -39.37% |
|  | p-value**^*^** | <0.001 |

**^*^**p-values based on Wilcoxon test

Abbreviations: BL, baseline; CI, confidence interval; FU2, end of trial; IL-6, interleukin-6.

**Supp. Table 9.** Linear regression estimates of the change in inflammatory biomarker levels due to vitamin D3 supplementation at the end of trial (n = 115).

| Model 1  (Unadjusted) | β-coefficient (95% CI) | **-0.72 (-1.21; -0.23)** |
| --- | --- | --- |
|  | Percentage Actual Change from BL to FU2 (95% CI)**^1^** | **-39.3 (-56.8; -14.7)** |
|  | p-value | <0.001 |
| Model 2  (Adjusted) | β-coefficient (95% CI) | **-0.91 (-1.41; -0.40)** |
|  | Percentage Actual Change from BL to FU2 (95% CI)**^1^** | **-46.8 (-62.4; -24.2)** |
|  | p-value | <0.001 |

Notes: linear regression models were used to estimate IL-6 biomarker changes in the intervention group versus placebo group (reference); Model 1 was unadjusted, Model 2 was adjusted for baseline log_2_ IL-6 concentration (continuous), baseline age (continuous), sex, baseline serum 25(OH)D (continuous), BMI (continuous), cancer stage (I, II, III, or IV), time since surgery (No surgery, 0-1, 2-3, 4-6, 7-9, 10-12, >12 months), previous chemotherapy and previous radiotherapy.

**Supp. Table 10.** Comparison of Interleukin-6 status by treatment group at baseline and end-of-trial

| **Treatment Group** | **Low** (n/%) | **High** (n/%) | **p-value (Chi²)** |
| --- | --- | --- | --- |
| **Baseline** |  |  |  |
| Placebo (n = 60) | 41 (68.3) | 19 (31.7) | 0.610 |
| Vitamin D (n = 55) | 40 (72.7) | 15 (27.3) |  |
|  |  |  |  |
| **Follow-up** |  |  |  |
| Placebo (n = 60) | 43 (71.7) | 17 (28.3) | 0.019 |
| Vitamin D (n = 55) | 49 (89.9) | 6 (10.9) |  |

Notes: Interleukin-6 was categorized as low (≤7pg/mL) or high (>7pg/mL).

**Supp. Table 11.** Exploratory linear regression estimates of the effects of vitamin D3 supplementation on inflammatory biomarkers at the end of trial (Intention-To-Treat Analysis, n=126).

| **Abbreviation** | **Biomarker name** | **β (s.e)** | **P-value** |
| --- | --- | --- | --- |
| 4E-BP1 | Eukaryotic translation initiation factor 4E-binding protein 1 | -0.15 (0.22) | 0.484 |
| ADA | Adenosine Deaminase | -0.03 (0.11) | 0.799 |
| AXIN1 | Axin-1 | -0.11 (0.16) | 0.503 |
| CASP-8 | Caspase-8 | -0.07 (0.20) | 0.743 |
| CCL11 | Eotaxin | -0.12 (0.06) | 0.053 |
| CCL19 | C-C motif chemokine 19 | 0.05 (0.09) | 0.583 |
| CCL20 | C-C motif chemokine 20 | -0.26 (0.20) | 0.196 |
| CCL23 | C-C motif chemokine 23 | 0.04 (0.07) | 0.496 |
| CCL25 | C-C motif chemokine 25 | 0.02 (0.07) | 0.738 |
| CCL28 | C-C motif chemokine 28 | -0.13 (0.07) | 0.062 |
| CCL3 | C-C motif chemokine 3 | -0.20 (0.13) | 0.128 |
| CCL4 | C-C motif chemokine 4 | -0.03 (0.10) | 0.776 |
| CD244 | Natural killer cell receptor 2B4 | -0.04 (0.06) | 0.480 |
| CD40 | CD40L receptor | -0.02 (0.07) | 0.820 |
| CD5 | T-cell surface glycoprotein CD5 | -0.10 (0.06) | 0.087 |
| CD6 | T cell surface glycoprotein CD6 isoform | -0.08 (0.09) | 0.403 |
| CD8A | T-cell surface glycoprotein CD8 alpha chain | -0.06 (0.09) | 0.536 |
| **CDCP1** | **CUB domain-containing protein 1** | **-0.17 (0.08)** | **0.034** |
| CSF-1 | Macrophage colony-stimulating factor 1 | -0.06 (0.03) | 0.080 |
| CST5 | Cystatin D | -0.04 (0.06) | 0.573 |
| CX3CL1 | Fractalkine | -0.04 (0.07) | 0.509 |
| CXCL1 | C-X-C motif chemokine 1 | 0.02 (0.10) | 0.874 |
| **CXCL11** | **C-X-C motif chemokine 11** | **-0.27 (0.13)** | **0.042** |
| CXCL5 | C-X-C motif chemokine 5 | -0.08 (0.09) | 0.359 |
| **CXCL6** | **C-X-C motif chemokine 6** | **-0.21 (0.09)** | **0.023** |
| CXCL9 | C-X-C motif chemokine 9 | -0.02 (0.13) | 0.850 |
| DNER | Delta and Notch-like epidermal growth factor-related receptor | -0.03 (0.04) | 0.532 |
| EN-RAGE | Protein S100-A12 | 0.02 (0.21) | 0.918 |
| FGF-19 | Fibroblast growth factor 19 | -0.01 (0.21) | 0.943 |
| FGF-21 | Fibroblast growth factor 21 | -0.10 (0.22) | 0.655 |
| FGF-23 | Fibroblast growth factor 23 | -0.09 (0.20) | 0.725 |
| FGF-5 | Fibroblast growth factor 5 | -0.06 (0.05) | 0.292 |
| Flt3L | Fms-related tyrosine kinase 3 ligand | -0.06 (0.06) | 0.313 |
| HGF | Hepatocyte growth factor | -0.02 (0.07) | 0.766 |
| IL-10 | Interleukin-10 | 0.03 (0.09) | 0.721 |
| IL-10RA | Interleukin-10 receptor subunit alpha | -0.02 (0.06) | 0.800 |
| IL-10RB | Interleukin-10 receptor subunit beta | -0.02 (0.04) | 0.567 |
| IL-12B | Interleukin-12 subunit beta | -0.02 (0.08) | 0.760 |
| IL-15RA | Interleukin-15 receptor subunit alpha | 0.01 (0.05) | 0.912 |
| IL-17C | Interleukin-17C | 0.07 (0.20) | 0.711 |
| IL-18 | Interleukin-18 | 0.00 (0.09) | 0.994 |
| IL-18R1 | Interleukin-18 receptor 1 | 0.02 (0.08) | 0.789 |
| IL-7 | Interleukin-7 | 0.04 (0.11) | 0.718 |
| IL-8 | Interleukin-8 | 0.11 (0.28) | 0.701 |
| LAP TGF-beta-1 | Latency-associated peptide transforming growth factor beta-1 | 0.01 (0.07) | 0.920 |
| LIFR | Leukemia inhibitory factor receptor | -0.05 (0.05) | 0.297 |
| MCP-1 | Monocyte chemotactic protein 1 | -0.08 (0.07) | 0.298 |
| MCP-2 | Monocyte chemotactic protein 2 | -0.02 (0.07) | 0.830 |
| MCP-3 | Monocyte chemotactic protein 3 | -0.09 (0.23) | 0.692 |
| MCP-4 | Monocyte chemotactic protein 4 | -0.11 (0.09) | 0.226 |
| MMP-10 | Matrix metalloproteinase-10 | -0.04 (0.09) | 0.682 |
| OPG | Osteoprotegerin | -0.10 (0.05) | 0.058 |
| OSM | Oncostatin-M | 0.13 (0.18) | 0.457 |
| PD-L1 | Programmed cell death 1 ligand 1 | -0.03 (0.06) | 0.614 |
| SCF | Stem cell factor | -0.04 (0.07) | 0.591 |
| SIRT2 | SIR2-like protein 2 | -0.10 (0.22) | 0.638 |
| SLAMF1 | Signaling lymphocytic activation molecule | 0.00 (0.07) | 0.976 |
| ST1A1 | Sulfotransferase 1A1 | -0.02 (0.15) | 0.887 |
| STAMBP | STAM-binding protein | -0.01 (0.16) | 0.954 |
| TGF-alpha | Transforming growth factor alpha | 0.14 (0.11) | 0.202 |
| TNF | Tumor necrosis factor | -0.03 (0.08) | 0.660 |
| TNFB | TNF-beta | -0.02 (0.06) | 0.667 |
| TNFRSF9 | Tumor necrosis factor receptor superfamily member 9 | -0.01 (0.06) | 0.834 |
| TNFSF14 | Tumor necrosis factor ligand superfamily member 14 | 0.04 (0.13) | 0.740 |
| TRAIL | TNF-related apoptosis-inducing ligand | -0.01 (0.06) | 0.868 |
| TRANCE | TNF-related activation-induced cytokine | 0.12 (0.11) | 0.311 |
| TWEAK | Tumor necrosis factor (Ligand) superfamily, member 12 | 0.01 (0.06) | 0.880 |
| uPA | Urokinase-type plasminogen activator | -0.05 (0.05) | 0.359 |
| VEGF-A | Vascular endothelial growth factor-A | -0.05 (0.08) | 0.564 |

**^1^**Biomarker concentrations are in NPX values; ; Biomarkers in bold have p-values < 0.05 after adjustment for baseline concentration of the respective inflammatory biomarker (continuous), baseline age (continuous), sex, baseline serum 25(OH)D (continuous), BMI (continuous), cancer stage (I, II, III, or IV), time since surgery (No surgery, 0-1, 2-3, 4-6, 7-9, 10-12, >12 months), previous chemotherapy and previous radiotherapy.

Abbreviations: s.e, standard error.
